# Comparative Effects of Bacterial and Yeast Fermentation on Nutritional & Bioactive Properties in Chickpea Protein Isolate

**DOI:** 10.1002/fsn3.72099

**Published:** 2026-07-20

**Authors:** Bilgen Özsoy, Buse Bıyıklı, Cansu Yay, Özlem Aslan, Emine Aytunga Arık Kibar, Müge İşleten Hoşoğlu

**Affiliations:** ^1^ Gebze Technical University Institute of Biotechnology Gebze/Kocaeli Türkiye; ^2^ Food Technologies Research Group The Scientific and Technological Research Council of Türkiye (TÜBİTAK) Marmara Research Center Gebze/Kocaeli Türkiye

**Keywords:** amino acid profile, *Bacillus subtilis*, *Kluyveromyces marxianus*, *Lactobacillus helveticus*, sustainability

## Abstract

Microbial fermentation of legume proteins and legume‐based ingredients is increasingly recognized as an effective strategy for developing innovative food products with improved nutritional and functional properties. Accordingly, this study aimed to comparatively evaluate the effects of fermenting chickpea protein isolate (CPI) using the lactic acid bacterium *Lactobacillus helveticus* , the bacterium *Bacillus subtilis* , and the yeast *Kluyveromyces marxianus* on its antioxidant capacity, nutritional quality, and protein molecular weight distribution. Microbial growth occurred predominantly within the first 24 h and differed significantly among microorganisms, with *B. subtilis* (2.7 ± 0.1 log CFU/mL increase) showing the highest and *L. helveticus* (0.9 ± 0.1 log CFU/mL increase) showing the lowest increase. Despite the reduction in certain amino acids, fermentation increased the essential‐to‐total amino acid ratio in all samples, with the greatest enhancement observed in *Bacillus*–fermented products. While antioxidant activity markedly enhanced following *B. subtilis* fermentation in DPPH, ABTS, and CUPRAC assays, *K. marxianus* and *L. helveticus* did not induce significant changes compared to the control sample, with total phenolic content remaining statistically unchanged across all groups. Fermentation also reduced phytic acid content from 44.5 μg/100 mL in the control sample to 38.3 μg/100 mL (14%) with *B. subtilis* , 30.5 μg/100 mL (31%) with *K. marxianus*, and most markedly to 13.3 μg/100 mL (70%) with *L. helveticus* , while condensed tannins exhibited only minor reductions, decreasing from 3.20 to 2.68 mg catechin/100 mL.

## Graphical abstract

Fermentation of chickpea protein isolate (> 95% protein) with *Kluyveromyces marxianus*, *Lactobacillus helveticus* , and *Bacillus subtilis* for 48 h modified its nutritional and functional properties. Fermentation affected soluble protein content and protein profile, amino acid composition, anti‐nutritional factors, and bioactive properties. These findings demonstrate that microbial fermentation is an effective strategy for improving the nutritional quality and bioactivity of chickpea protein isolates.

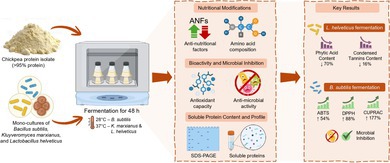

## 1 Introduction

The increasing global population has intensified the demand for sustainable, affordable, and high‐quality protein sources, shifting the focus from animal‐based to plant‐based alternatives. Chickpea ( *Cicer arietinum* L.) is an annual legume that ranks as the third most important crop globally in terms of total production, providing a valuable source of high‐quality dietary protein, along with beneficial carbohydrates, dietary fiber, and essential minerals (Arik Kibar and Aslan 2024; Liu et al. 2023). Chickpea protein isolates (CPI), which contain a minimum of 80% protein on a dry weight basis, are increasingly utilized as functional ingredients in the food industry owing to their nutritional profile and techno‐functional properties, such as emulsifying and foaming capacities (Grasso et al. 2022). However, the broader utilization of legume proteins remains limited by the presence of anti‐nutritional factors (ANFs) such as phytic acid and tannins, potential allergenicity, reduced mineral bioavailability, and suboptimal sensory characteristics compared to animal‐derived proteins (Liu et al. 2023; Nikbakht Nasrabadi et al. 2021).

Fermentation is a well‐established biotechnological strategy for improving the nutritional, functional, and sensory qualities of legume‐based ingredients (Alrosan et al. 2023; Fan et al. 2025). Fermentation can reduce ANFs, enhance mineral bioavailability, and improve protein digestibility through the activity of endogenous and microbial enzymes (Arbab Sakandar et al. 2023; Emkani et al. 2022; Sanjukta and Rai 2016). The selection of appropriate microbial strains plays a critical role in determining fermentation outcomes, as different microorganisms exhibit distinct metabolic capabilities that directly influence the chemical and nutritional properties of the final product (El Youssef et al. 2020; De Melo Pereira et al. 2020). Lactic acid bacteria (LAB), such as *Lactobacillus* species, are widely used for their ability to reduce ANFs and improve protein digestibility (Du et al. 2024; Emkani et al. 2022; Liu et al. 2023). *Bacillus* species are also known for their strong proteolytic activity, which can be beneficial for plant‐based protein modifications (Li and Wang 2021; Sahin et al. 2022; Zhang et al. 2021). Yeasts such as *Kluyveromyces marxianus*, although extensively used in flavor development and metabolite production, remain largely unexplored as single starter cultures for the fermentation of legume protein isolates, despite their potential contribution to the formation of bioactive compounds and amino acid release (El Youssef et al. 2020; Sahin et al. 2022).

While fermentation of chickpea‐based matrices using LAB and *Bacillus* species has been previously reported, studies focusing specifically on chickpea protein isolates are scarce, and yeast‐driven fermentations are virtually absent from literature. This study aimed to investigate the effects of fermentation of chickpea protein isolate (CPI) using *Lactobacillus helveticus* , *Bacillus subtilis* , and *Kluyveromyces marxianus*. The microbial strains used in this study were selected based on both our previous studies (Kurt et al. 2023; Sahin et al. 2022) and their well‐documented functionalities in fermentation systems (Meinlschmidt et al. 2016; Plessas et al. 2008). In addition, the selection was designed to represent different microbial groups (LAB, proteolytic bacteria, and yeast) to comparatively evaluate their distinct impacts on the modification of CPI. The control sample was compared with the fermented samples in terms of bioactive properties (antioxidant, antimicrobial, and total phenolic content), nutritional value (total amino acid profile and anti‐nutritional factors), and protein molecular weight distribution (SDS‐PAGE). This study provides a systematic comparison of different microbial fermentations of CPI, highlighting the importance of microbial selection in the development of fermented chickpea‐based functional ingredients.

## 2 Materials and Methods

### 2.1 Materials

Chickpea protein isolate (CPI) was prepared by TÜBİTAK Food Technologies Research group using a conventional extraction method, as described in a previous study (Arik Kibar and Aslan 2024). *Bacillus subtilis* (B‐3387), *Lactobacillus helveticus* (B‐4526), and *Kluyveromyces marxianus* (Y‐329) were obtained from NRRL Culture Collection (USDA Agricultural Research Service, IL, USA). All chemicals used in this study were of analytical grade unless otherwise indicated.

### 2.2 Preparation of Fermentation Media and Microbial Growth Conditions

Fermentation of CPI powder was performed for 48 h using *B. subtilis* , *K. marxianus* (both at 28°C, 130 rpm), and *L. helveticus* (at 37°C, 100 rpm) according to a previous study with some modifications (Kurt et al. 2023). Briefly, the cultures that were propagated twice until the late exponential growth phase (approximately 16–18 h) were centrifuged to collect the cells at 6000 rpm for 10 min at 4°C. A CPI solution (3%, w/w) was prepared at pH 6.5 for the growth of the strains. The CPI solution was sterilized at 121°C for 15 min to eliminate endogenous microbiota. The suspension was supplemented with 2% (w/v) glucose to enhance microbial growth. The CPI suspensions were inoculated with the strains from precultures (1%, w/v) at a concentration of 10^6–7^ colony‐forming units (CFU) per mL. Fermentation was terminated by adjusting the pH of the fermentation broth to 8.0 with 1 M NaOH to stop the activity of the microorganisms. This enabled the solubility of proteins that may have precipitated during fermentation while avoiding thermal treatment to preserve protein functionality (Arik Kibar and Aslan 2024; Özsoy et al. 2025). Then, the broths were incubated for 30 min under strain‐specific conditions and centrifuged at 7500 rpm for 10 min at 4°C to separate the cells from the suspension. The supernatants were stored at −20°C for further analysis. An uninoculated CPI solution was used as the control group (unfermented) and it was subjected to the same procedures as the fermented samples. Fermentations were performed in triplicates. All fermentations were monitored by determining the viable cell counts of cultures at times 0 (directly after inoculation), 24 h, and 48 h using the pour plate technique. After the incubation period, the results were expressed as log CFU/mL.

### 2.3 Determination of Total Soluble Protein Content

The soluble protein content of both fermented and control samples was determined according to the method of Lowry et al. (1951). The measurements were performed using a UV–Vis spectrophotometer (Shimadzu UV‐1280, Japan) and the results were expressed in milligram per milliliter (mg mL^−1^).

### 2.4 Sodium Dodecyl Sulfite‐Polyacrylamide Gel Electrophoresis (SDS‐PAGE)

SDS‐PAGE analysis was performed according to Laemmli (1970) using 12% separating gel and 6% stacking gel. Before electrophoresis, the soluble protein content in the supernatants was quantified using the method of Lowry et al. (1951) to ensure uniform protein loading into the wells. Then, the supernatants were mixed with sample buffer (pH 6.8 Tris, glycerol, bromophenol blue, SDS, and ß‐mercaptoethanol). The solutions were subjected to a quick spin and heated at 100°C for 5 min before electrophoresis. Twenty microliters of solution were loaded onto each well. The proteins were resolved at 500 mA for 15 min and then at a constant current of 923 mA for 1 h. The bands were stained for 30 min with 0.25 g L^−1^ Coomassie Brilliant Blue G‐250 (Fisher Scientific BP100‐25), 125 mL L^−1^ methanol, and 25 mL L^−1^ acetic acid solution and washed overnight with de‐staining solution containing 100 mL L^−1^ methanol and 100 mL L^−1^ acetic acid to remove the excess dye. A molecular weight marker ranging from 10.5 to 175.0 kDa (Opti‐Protein Marker G‐252; Applied Biological Materials Inc., Canada) was used as a standard.

### 2.5 Determination of Total Amino Acid Composition

The amino acid composition was determined following the procedure described by Wang et al. (2020) with minor adjustments to accommodate the specific characteristics of the samples. Approximately 0.5 g of supernatant obtained from fermented CPI and control samples was subjected to acid hydrolysis using 20 mL of 6 M HCl and incubated under sealed conditions at 110°C for 24 h. Following acid hydrolysis, the hydrolysates were cooled to room temperature and filtered through filter paper. No additional deproteinization was applied. An aliquot of 0.2 mL of the filtrate was transferred into a glass tube and evaporated to dryness under a nitrogen stream at 50°C. The residue was then derivatized by adding 0.5 mL of an acetonitrile:methanol:triethylamine mixture (100:50:20, v/v/v) and 0.1 mL phenylisothiocyanate (PITC) derivatization reagent. The mixture was incubated at 40°C for 30 min. After derivatization, the reaction mixture was again evaporated to dryness under nitrogen at 40°C, reconstituted in 5 mL of 0.02 M ammonium acetate solution, and filtered through a 0.45 μm membrane filter. The resulting solution was analyzed directly, without further dilution, using an ultra‐performance liquid chromatography system (LC‐20, Shimadzu, Kyoto, Japan) equipped with a UV detector. Chromatographic separation was achieved using an ACE 5 C18 column (250 mm × 4.6 mm, 5 μm) maintained at 40°C. A gradient elution program was applied using solvent A (5 mM NaH_2_PO₄·2H_2_O, pH 6.9) and solvent B (analytical‐grade acetonitrile). The derivatized amino acids were detected at 254 nm.

### 2.6 Determination of Antimicrobial Activity

The antimicrobial activity was evaluated against different test microorganisms ( *E. coli* , *S. aureus* , and *B. cereus* ) using both agar well and disc diffusion methods. For the agar well diffusion method (Ghosh et al. 2008), the bacterial cultures that were activated in nutrient broth (37°C for *E. coli* and *S. aureus* and 25°C for *B. cereus*) for 18–24 h were adjusted to 0.5 McFarland standard turbidity. 100 μL of the active cultures were mixed with 20 mL of Brain Heart Infusion agar medium in petri dishes. In the solidified agar medium, 50 mL of supernatant was loaded into the open wells. Chloramphenicol solution (0.3 mg mL^−1^) and sterile distilled water were used as positive and negative controls, respectively. For the disc diffusion method (Celiktas et al. 2007), 100 mL of the active cultures adjusted to 0.5 McFarland were spread on the surface of Mueller‐Hinton agar. Sterile blank discs (antimicrobial susceptibility test discs, Bioanalyse) that absorbed 15 μL of the supernatants were placed onto agar plates. Standard discs with chloramphenicol and sterile distilled water‐absorbed discs were used as positive and negative controls, respectively. The diameter of the inhibition zones was measured after 24 h of incubation time was completed.

### 2.7 Determination of Total Phenolic Content

The total phenolic content (TPC) was determined according to the method described by Singleton et al. (1999). For this purpose, 900 mL of distilled water was mixed with 100 mL of the supernatant. Then, 5 mL of 2 N Folin–Ciocalteu reagent and 4 mL of sodium carbonate (75 g/L) were added to the sample separately with mixing after each reagent addition. The samples were incubated for 2 h in the dark at room temperature. Measurements were performed at a wavelength of 765 nm using a spectrophotometer (Shimadzu UV‐1280, Japan). The TPC was calculated as mg gallic acid per 100 mL of sample using a calibration curve prepared with a gallic acid standard.

### 2.8 Determination of Antioxidant Activity

To evaluate the antioxidant activity of the fermented products, three assays were conducted: the 2,2‐diphenyl‐1‐picrylhydrazyl (DPPH) radical scavenging assay, the 2,2‐azinobis (3‐ethylbenzothiazoline‐6‐sulfonic acid) (ABTS) radical scavenging assay, and the cupric reducing antioxidant capacity (CUPRAC).

DPPH^●^ radical scavenging activity was evaluated according to the procedure described by Brand‐Williams et al. (1995) with minor modifications. For each fermentation, 500 μL of sample was mixed with 500 μL of 0.2 mM DPPH solution and kept in the dark for 45 min. The absorbance was measured using a UV–Vis spectrophotometer (Shimadzu UV‐1280) at a wavelength of 515 nm. As a blank, a solution containing methanol and deionized water was used and subtracted from the absorbance of samples. The DPPH scavenging activity in percentage was calculated using Equation (1). The results are expressed in mg Trolox equivalent (TE) per 100 mL of sample.

(1) $$\text{DPPH scavenging activity}\;\left(\%\right)=\frac{\left(A_{\text{DPPH}}-A_{\text{sample}}\right)}{A_{\text{DPPH}}}\times 100$$

where A_DPPH_ is the absorbance of the control containing radical solution and methanol, and A_sample_ is the absorbance of sample solution.

The ABTS^●+^ radical scavenging assay was performed according to Re et al. (1999) with some modifications. A mixture of solutions (7 mM ABTS solution and 12.25 mM potassium persulfate solution) was prepared in a flask and was stored at room temperature in the dark for 12–16 h to stabilize. Prior to analysis, the radical solution was diluted with 0.1 M phosphate‐buffered saline (PBS, pH 7.7) to achieve an absorbance of 0.70 ± 0.02 at 734 nm. The absorbance values were recorded every minute for a total of six minutes using a spectrophotometer (Shimadzu UV‐1280). The percentage inhibition was calculated using Equation (2), and the results were expressed in mM TE per mL of sample.

(2) $$\text{ABTS scavenging activity}\;\left(\%\right)=\frac{\left(A_{\text{ABTS}}-A_{\text{sample}}\right)}{A_{\text{ABTS}}}\times 100$$

where A_ABTS_ and A_sample_ are the absorbance of the ABTS radical solution and sample solutions, respectively.

The CUPRAC assay was performed according to the method reported by Apak et al. (2010), with some modifications. A 0.01 M CuCl_2_ solution was prepared by dissolving 0.067 g CuCl_2_ in 50 mL distilled water, a 7.5*10^−3^ M neocuproine solution was prepared by dissolving 0.078 g neocuproine in 50 mL 96% ethanol, and finally, 1.0 M ammonium acetate buffer at pH 7.0 was prepared by dissolving 3.854 g ammonium acetate in 50 mL distilled water. Then, 1 mL of each of these three solutions was placed in a test tube, and 125 μL of the sample was added, followed by 975 μL of distilled water, and kept in the dark for 30 min. Absorbance values were measured at a wavelength of 450 nm using a spectrophotometer (Shimadzu UV‐1280, Japan), and the results were expressed in mg TE per 100 mL of sample.

### 2.9 Evaluation of Anti‐Nutritional Properties

The phytic acid content of all samples was determined according to the colorimetric method of Haug and Lantzsch (1983) with minor modifications. Briefly, 0.1 g of supernatant obtained from fermented CPI and control samples was extracted with 10 mL of Na_2_SO_4_ solution prepared in 0.4 M HCl by shaking at 250 rpm for 2 h, followed by centrifugation at 4600 rpm for 20 min. An aliquot (1 mL) of the supernatant was transferred into two parallel screw‐cap test tubes, mixed with 2 mL of ferric reagent (ammonium iron (III) sulfate dodecahydrate dissolved in 2 N HCl), vortexed, and incubated in a boiling water bath at 95°C for 30 min. After cooling in an ice bath for 15 min and equilibrating to room temperature, the samples were centrifuged again at 4600 rpm for 10 min. Subsequently, 1 mL of the clarified supernatant was mixed with 3 mL of 1% (w/v) 2,2′‐bipyridine solution containing thioglycolic acid, and the absorbance was measured at 519 nm after 45 s using a UV–Vis spectrophotometer. Sodium phytate (C_6_H_6_O_24_P_6_Na_12_) was used as the reference standard, and the phytic acid content was calculated and expressed as mg per 100 g of sample.

The condensed tannin content of the samples was determined using the colorimetric vanillin–HCl method described by Price et al. (1980). Briefly, 0.2 g of supernatant obtained from fermented CPI and control samples was extracted with 10 mL of 100% methanol by shaking for 15 min, followed by incubation in a water bath at 30°C for 15 min and centrifugation at 9000 rpm for 10 min. An aliquot (1 mL) of the supernatant was transferred into two parallel test tubes, and 5 mL of freshly prepared vanillin reagent (1% vanillin in methanol mixed 1:1 with 8% HCl in methanol) was added to each. For blank measurements, 5 mL of 4% HCl in methanol was used instead of the vanillin. After incubation at room temperature for 30 min, the absorbance was measured at 500 nm using a UV–Vis spectrophotometer. Catechin was used as the reference standard, and tannin content was calculated and expressed as mg catechin equivalents per 100 g sample.

### 2.10 Statistical Analysis

Experimental results were mean values of triplicate analyses (*n* = 3). All data were analyzed using one‐way analysis of variance (ANOVA) at a 95% confidence level. Post hoc comparisons were conducted using Tukey's Honestly Significant Difference (HSD) test to identify significant differences between the groups (*p* < 0.05). Statistical analyses were performed using the JMP Pro software (version 18.0.2, SAS Institute Inc., Cary, NC, USA).

## 3 Results and Discussion

### 3.1 Viable Cell Counts and Acidifications

**FIGURE 1:**
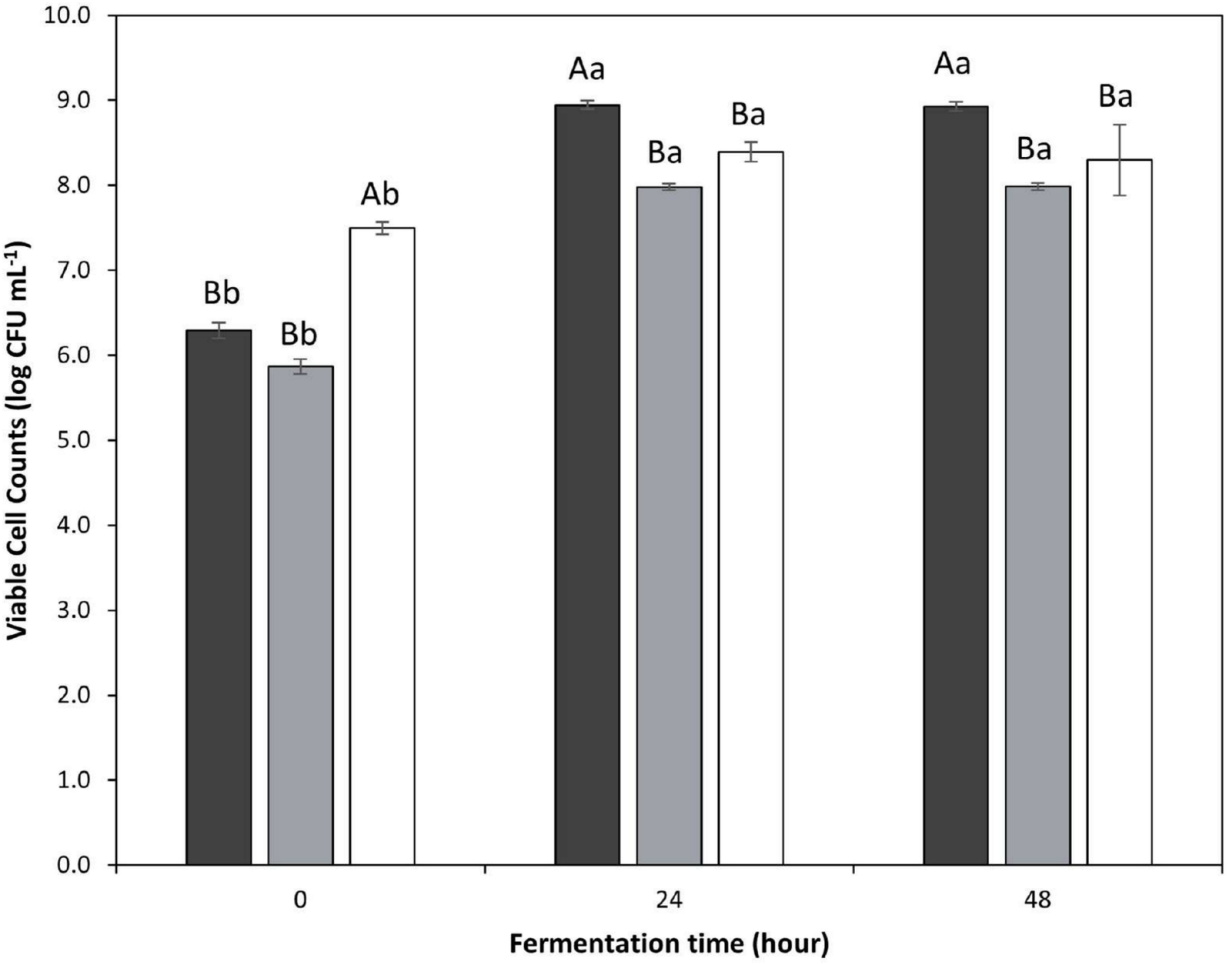
Microbial growth of *Bacillus subtilis* (B‐3387, 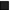), *Kluyveromyces marxianus* (Y‐329, 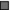) and *Lactobacillus helveticus* (B‐4526, 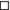) on chickpea protein isolate (CPI) medium in terms of viable cell counts (log CFU mL^−1^) during fermentation (0, 24, and 48 h). Each bar represents the mean of three replicates with error bars indicating the standard deviation. ^A,B^Uppercase letters denote significant differences among the different microorganisms evaluated at the same time (*p* < 0.01). ^a,b^Lowercase letters indicate significant differences between fermentation hours within the same microorganism (*p* < 0.01).

The viable cell count results demonstrated that all tested microorganisms could grow in CPI medium during fermentation, confirming that CPI could support microbial metabolism with an additional carbon source (Figure 1). For all the tested strains, substantial growth was observed after 24 h of fermentation. However, the extent of growth varied significantly according to the microbial species (*p* < 0.001). Among the tested species, *B. subtilis* exhibited the highest growth increase (2.7 ± 0.1 log CFU/mL) after 24 h of incubation. This apparent increase is consistent with previous studies reporting the strong adaptability of *Bacillus* species to protein‐rich substrates, primarily because of their high extracellular proteolytic activity, which enables efficient utilization of proteins and carbon sources (Li and Wang 2021; Zhang et al. 2021; Kurt et al. 2023). Additionally, *K. marxianus* showed moderate growth increase (2.1 ± 0.1 log CFU/mL) in CPI medium, which aligns with the literature describing its ability to utilize amino acids and peptides as nitrogen sources, likely contributing to the observed growth (El Youssef et al. 2020). In contrast, the lowest growth was recorded in *L. helveticus* , with an increase of 0.9 ± 0.1 log CFU/mL after 24 h.

The pH of the medium is a critical parameter that influences microbial growth kinetics, enzyme activity, and metabolite formation during fermentation (Kurt et al. 2023). In the present study, the initial pH of CPI medium at the onset of fermentation was approximately 6.5, which is within the optimal range for the growth of most microorganisms. A slight increase in pH was observed between 24 and 48 h in CPI fermented with *B. subtilis* (pH 5.0 and 5.5, respectively), whereas *K. marxianus* fermentation resulted in a moderate pH decrease over the same period (pH 5.9 and 5.6, respectively) (Figure 1). The increase of pH in *Bacillus* fermentation could be associated with proteolytic activity and the release of alkaline metabolites. In contrast, fermentation with *L. helveticus* led to a pronounced and progressive pH reduction, reaching a pH 4.3 after 24 h and further decreasing to a pH of 4.0 after 48 h. This behavior is characteristic of lactic acid bacteria, which convert available fermentable substrates into lactic acid as their primary metabolic end products (Emkani et al. 2022; Liu et al. 2023).

No significant differences (*p* > 0.05) in microbial growth were observed between 24 and 48 h for any of the tested microorganisms. This indicates that the cultures had likely entered the stationary phase following the exponential growth observed during the first 24 h. This behavior could also be associated with the pH changes observed during fermentation. The acidification during the early stages, mostly due to organic acid production, may have contributed to limitation of further microbial proliferation. Meinlschmidt et al. (2016) and Tonini et al. (2024) also demonstrated that pH fluctuations can significantly influence cell density.

### 3.2 Analysis of Protein Fragmentations by SDS‐PAGE

**FIGURE 2:**
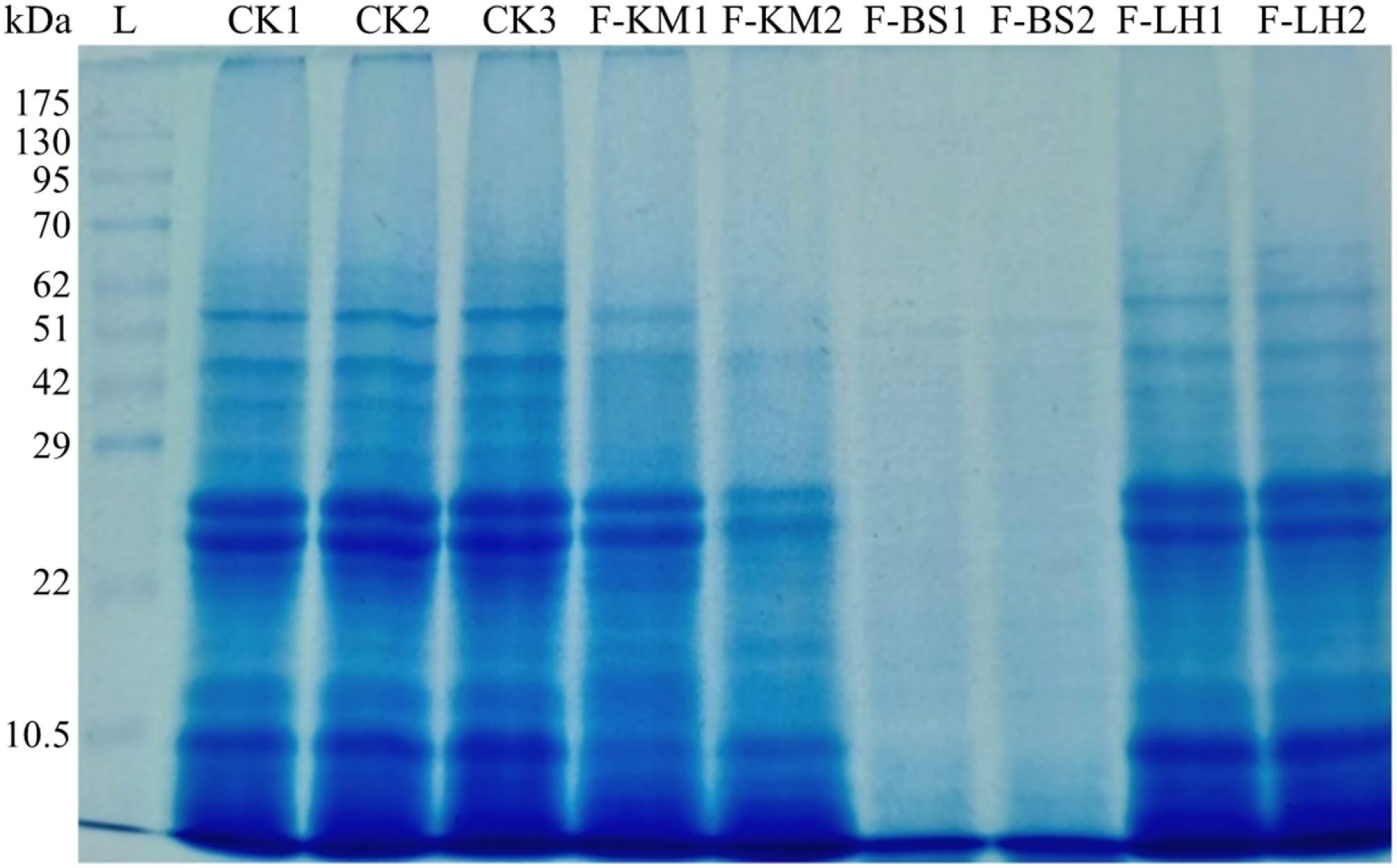
SDS‐PAGE analysis of chickpea protein isolate (CPI) samples fermented for 48 h by *Bacillus subtilis* (B‐3387), *Kluyveromyces marxianus* (Y‐329), and *Lactobacillus helveticus* (B‐4526) (L: Ladder, molecular weight marker, with sizes in kDa indicated on the left; F‐KM1, F‐KM2: Replicates for *K. marxianus* fermentations; F‐BS1, F‐BS2: Replicates for *B. subtilis* fermentations; F‐LH1, F‐LH2: Replicates for *L. helveticus* fermentations) in duplicates and control samples (CK1, CK2, and CK3 in triplicates).

Differences in proteolysis profiles were observed with different microorganisms after 48 h of fermentation, as shown by the SDS‐PAGE profile, which enabled the visualization of soluble proteins ranging from 175 to 10.5 kDa in this study (Figure 2). For the control groups, several bands with corresponding molecular weights in the ranges of < 75 kDa and < 10.5 kDa were visible, allowing the observation of several bands at ~51–70, 42–51, 25–40, and below 10.5 kDa. The control sample exhibited distinct bands corresponding to legumin α (36–40 kDa), legumin β (20–25 kDa), and vicilins (~50 kDa), as reported in previous studies on chickpeas (Arik Kibar and Aslan 2024; Di Francesco et al. 2024). Bands observed in the ~51–70 kDa region may correspond to chickpea globulins, where proteins around ~70 kDa are commonly assigned to convicilin, whereas bands near ~60 kDa and~50 kDa are frequently attributed to legumin monomers and vicilin subunits, respectively (Di Francesco et al. 2024). Compared to the control, these bands became visibly weaker in samples fermented with *K. marxianus* and were almost completely absent in *Bacillus*‐fermented samples. The pronounced disappearance of almost all bands in *Bacillus*‐fermented CPI solution can be attributed to the strong extracellular proteolytic activity of *Bacillus* species, which enables the extensive hydrolysis of major storage proteins into lower‐molecular‐weight peptides and amino acids. This observation is consistent with the high viable cell counts and substantial reduction in specific amino acid groups observed in *Bacillus*‐fermented samples such as the branched‐chain amino acids (Val, Leu, Ile) and aromatic amino acids (Phe, Tyr), indicating intensive protein utilization and degradation (Kurt et al. 2023; Sanjukta and Rai 2016). Notable protein degradation was also consistent with the high viable cell counts recorded for *B. subtilis* and the relatively moderate pH decrease, conditions known to favor protease activity rather than acid‐induced protein aggregation. In contrast, fermentations using *K. marxianus* and *L. helveticus* induced milder hydrolysis, consistent with their limited proteolytic capacities under similar fermentation conditions (Emkani et al. 2022).

### 3.3 Total Amino Acid Composition

Fermentation markedly altered the total amino acid composition of the CPI medium, and all microbial treatments resulted in a substantial decrease in the total amino acid content compared to the control group (Table 1). The total amino acid content decreased from 1087 mg/100 g (control) to 461 mg/100 g in the CPI medium fermented by *B. subtilis* , 667 mg/100 g in *K. marxianus* fermentation, and 582 mg/100 g in *L. helveticus* fermentation, indicating varying degrees of protein degradation and amino acid utilization among the microorganisms. This reduction is consistent with the proteolytic activities of fermenting strains, where amino acids may be utilized as carbon and nitrogen sources or metabolized into various metabolites during fermentation (Sanjukta and Rai 2016). Among the microorganisms, *B. subtilis* caused the most drastic reduction in total amino acid content, in agreement with its higher growth in this study (Figure 1) and its well‐documented extracellular protease system capable of rapid peptide and amino acid turnover (Gao et al. 2024). A pronounced decline in Asp and Glu, the two major acidic amino acids in chickpea proteins (Arik Kibar and Aslan 2024), was observed across all microbial treatments. As these amino acids are abundant in vicilin and legumin subunits, their reduction supports the SDS‐PAGE observation of storage protein breakdown (Figure 2).

**TABLE 1:** Amino acid composition of chickpea protein isolate (CPI) samples fermented for 48 h by *Bacillus subtilis* (B‐3387), *Kluyveromyces marxianus* (Y‐329), and *Lactobacillus helveticus* (B‐4526).

Amino acid (mg/100 g sample)	F‐BS	F‐KM	F‐LH	CK
Aspartic acid (Asp)	35 ± 1^b^	29 ± 1^b^	28 ± 3^b^	122 ± 2^a^
Glutamic acid (Glu)	66 ± 2^b^	80 ± 1^b^	59 ± 2^b^	235 ± 34^a^
Serine (Ser)	8 ± 1^c^	30 ± 0^a^	18 ± 3^b^	30 ± 0^a^
Glycine (Gly)	13 ± 0^b^	21 ± 3^b^	24 ± 5^ab^	38 ± 2^a^
Histidine (His)^†^	**5 ± 1** ^**a**^	**4 ± 1** ^**a**^	**4 ± 4** ^**a**^	**12 ± 2** ^**a**^
Arginine (Arg)	25 ± 1^b^	39 ± 1^b^	27 ± 3^b^	93 ± 8^a^
Threonine (Thr)	**24 ± 0** ^**a**^	**3 ± 1** ^**c**^	**2 ± 2** ^**c**^	**10 ± 1** ^**b**^
Alanine (Ala)	10 ± 1^b^	29 ± 1^a^	21 ± 4^ab^	34 ± 5^a^
Proline (Pro)	18 ± 1^b^	30 ± 1^ab^	25 ± 3^ab^	41 ± 7^a^
Tyrosine (Tyr)	0 ± 0^b^	57 ± 4^a^	56 ± 1^a^	59 ± 3^a^
Valine (Val)	**61 ± 2** ^**a**^	**69 ± 5** ^**a**^	**68 ± 0** ^**a**^	**74 ± 4** ^**a**^
Methionine (Met)	**N.D**.	**N.D**.	**N.D**.	**N.D**.
Isoleucine (Ile)	**13 ± 1** ^**b**^	**26 ± 5** ^**ab**^	**27 ± 4** ^**ab**^	**46 ± 8** ^**a**^
Leucine (Leu)	**57 ± 1** ^**b**^	**78 ± 6** ^**ab**^	**77 ± 4** ^**ab**^	**90 ± 6** ^**a**^
Phenylalanine (Phe)	**42 ± 1** ^**b**^	**65 ± 1** ^**ab**^	**53 ± 4** ^**ab**^	**76 ± 11** ^**a**^
Lysine (Lys)	**86 ± 3** ^**b**^	**110 ± 10** ^**ab**^	**97 ± 5** ^**b**^	**131 ± 2** ^**a**^
TEAA^‡^	287 ± 7	354 ± 27	327 ± 22	438 ± 29
TAA^‡^	461 ± 11^c^	667 ± 36^b^	582 ± 42^bc^	1087 ± 1^a^
TEAA/TAA (%)	62 ± 0	53 ± 1	56 ± 0	40 ± 3

*Note:* Results are expressed as mean ± standard deviation (*n* = 3). ^a‐c^Different superscript letters indicate significant differences within the same row (*p* < 0.05) following one‐way ANOVA (Tukey). Tryptophan content was not determined.

Abbreviations: CK, control group (unfermented); F‐BS, CPI fermented by *B. subtilis* ; F‐KM, CPI fermented by *K. marxianus*; F‐LH, CPI fermented by *L. helveticus* ; N.D.: Not detected.

^†^ Amino acids written in bold indicate essential amino acids.

^‡^ Represents total essential amino acids (TEAA) and total amino acids (TAA).

A significant reduction (*p* < 0.05) in branched‐chain amino acids (Val, Leu, Ile), aromatic amino acids (Phe, Tyr), and Lys was observed in *Bacillus*‐fermented sample compared to that in the control group (Table 1). In contrast, the decreases observed following *K. marxianus* and *L. helveticus* fermentation were considerably milder. These amino acids are important precursors of flavor‐active and bioactive compounds. Therefore, their partial reduction may indicate their conversion into microbial metabolites or their use in microbial metabolism (Wang et al. 2025). Threonine content was higher in the *Bacillus*‐fermented samples (24 mg/100 g) than in the control group (10 mg/100 g). As amino acid analysis was performed on the cell‐free supernatant, this observation likely reflects a relative enrichment of threonine‐containing soluble peptides/proteins in the medium rather than an accumulation of free threonine. Such enrichment may arise from the proteolytic specificity of *B. subtilis* , which can release threonine‐rich fragments into the soluble fraction, whereas other amino acids are more extensively metabolized. Methionine was undetectable in all samples, including the control group, which is consistent with the intrinsically low sulfur amino acid content of chickpeas and is supported by previous compositional studies on legumes (Arik Kibar and Aslan 2024). However, despite the reductions observed in specific amino acid groups, the ratio of total essential amino acids to total amino acids increased in all fermented samples compared to that in the control, with the highest increase detected in *Bacillus*–fermented products.

### 3.4 Antimicrobial Activity

**FIGURE 3:**
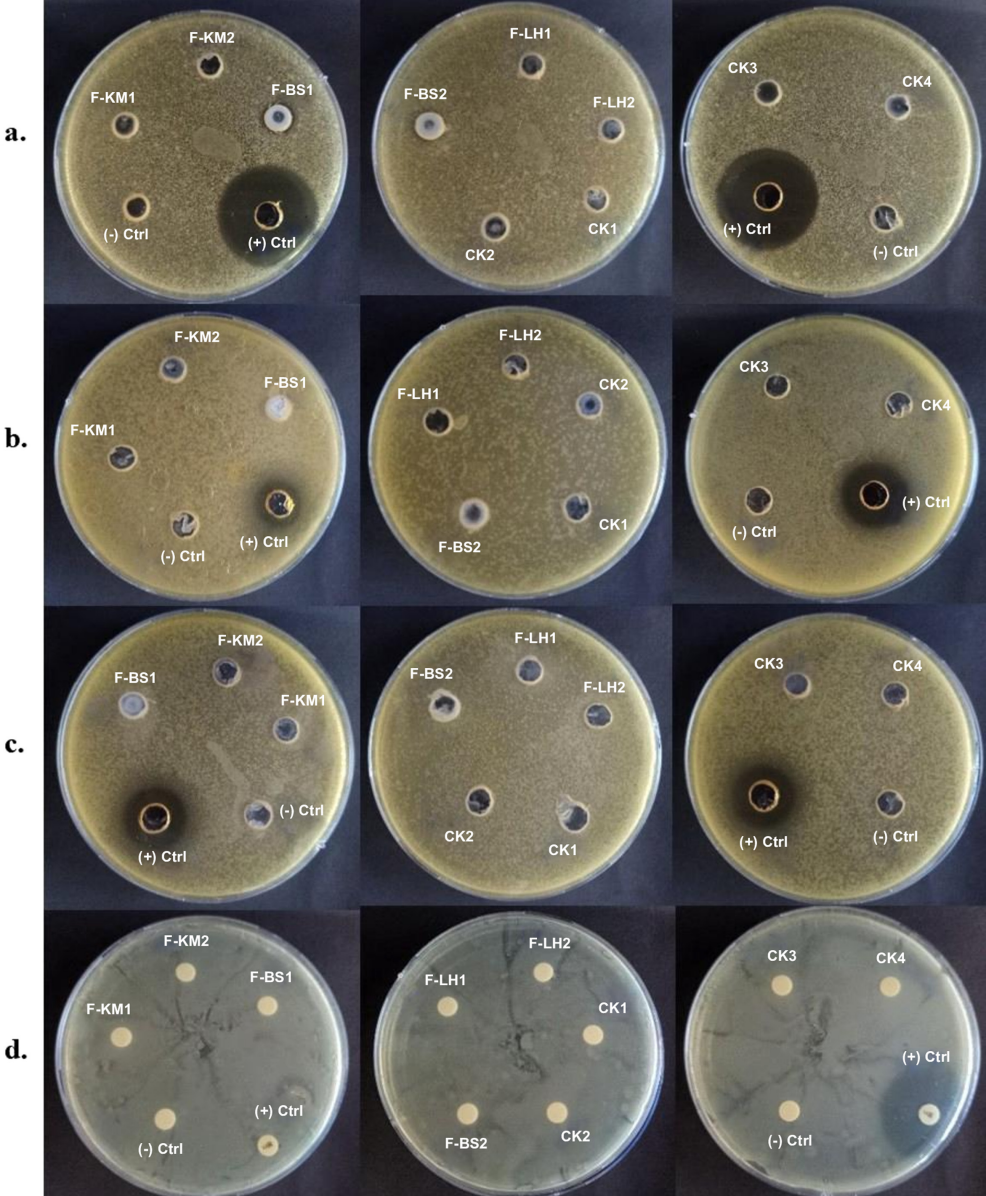
Antimicrobial activity of fermented chickpea protein isolates (F‐KM1, F‐KM2: Replicates for *K. marxianus* fermentation; F‐BS1, F‐BS2: Replicates for *B. subtilis* fermentation; F‐LH1, F‐LH2: Replicates for *L. helveticus* fermentation) and control group (CK1, CK2, CK3, and CK4) against *B. cereus* (a), *E. coli* (b, d), and *S. aureus* (c).

The antimicrobial activity of CPI samples, including the control and those fermented with *B. subtilis* , *L. helveticus* and *K. marxianus*, was evaluated based on their capacity to inhibit the growth of common foodborne pathogens, namely *B. cereus* , *E. coli* and *S. aureus* . As shown in Figure 3, CPI fermented by *B. subtilis* inhibited the growth of gram‐positive bacteria *B. cereus* , with a measured inhibition zone of 10 mm. This finding demonstrates that *B. cereus* is the most susceptible species to CPI solutions. A comparable observation was reported by Kanatt and Sharma (2011), who investigated the hull extracts of bengal gram, pigeon pea and mung beans. Among these, only the extracts from bengal gram and pigeon pea were reported to inhibit the growth of *B. cereus* , whereas no inhibitory activity was observed against *S. aureus* , *E. coli* , or *P. fluorescens* . This aligns with the results of the present study and underscores the notion that gram‐positive bacteria demonstrate heightened sensitivity to polyphenols and antioxidant compounds compared to gram‐negative bacteria (Salman et al. 2022). This tendency can be attributed to the distinct differences in the cell wall structure. In particular, gram‐negative bacteria possess an additional outer membrane that functions as a protective barrier and limits the penetration of high‐molecular weight molecules (G. Zhou et al. 2023).

### 3.5 Total Soluble Protein (TSP) Content

In the present study, the total soluble protein concentration of the control sample (14.0 mg mL^−1^) decreased significantly in all fermented samples (8.2–9.2 mg mL^−1^), with no differences observed among the microorganisms. Similar variability has been reported in legume protein fermentation studies, where soluble protein content may increase due to proteolysis and peptide release, remain unchanged, or even decrease depending on the substrate type, pH changes, and fermentation mode (Ankolekar et al. 2012; Yousefi et al. 2025). For example, during solid‐state fermentation of soybean meal, soluble protein increased from 3.31 to 10.16 g/100 g under optimized conditions, which is consistent with extensive proteolysis and improved solubility (Chotinu‐kul et al. 2025). Conversely, in a recent study, pea protein powder solubility decreased by approximately 20% after lactic acid bacterial fermentation, despite minimal changes in several other functional traits (Kaleda et al. 2025). Similarly, lactic acid fermentation of pea protein isolates has been reported to reduce soluble protein content to approximately 66% of the control after fermentation, indicating that acid‐driven aggregation can outweigh any solubilizing effect from partial proteolysis (Shi et al. 2021). This interpretation is further supported by the pronounced acidification observed in this study during *L. helveticus* fermentation (pH ~4.0), which is close to the isoelectric point of legume globulins and therefore promotes protein aggregation and reduces solubility. In addition, the extensive disappearance of medium‐molecular‐weight bands in the SDS‐PAGE profiles of Bacillus–fermented samples indicated intensive proteolysis and microbial utilization of soluble nitrogen, which probably contributed to the observed decrease in total soluble protein despite enhanced peptide formation.

### 3.6 Antioxidant Activity and Total Phenolics Content (TPC)

The antioxidant activity of all samples was evaluated to investigate the potential of fermentation‐derived metabolites in improving the antioxidant activity of products. As shown in Table 2, fermentation significantly affected the antioxidant activity of CPI. The DPPH assay showed that *B. subtilis* fermentation led to a 1.88‐fold increase in antioxidant capacity (0.75 mg TE/100 mL) compared to that of control (0.40 mg TE/100 mL). In contrast, *K. marxianus* (0.44 mg TE/100 mL) did not show a significant difference from the control (*p* > 0.05), whereas *L. helveticus* exhibited an intermediate effect, with a 1.38‐fold increase in antioxidant activity (0.55 mg TE/100 mL). Similarly, fermentation with *B. subtilis* also demonstrated the highest activity in ABTS (2.26 mM TE/mL sample; 1.54‐fold increase) and CUPRAC (24.76 mg TE/100 mL; 2.77‐fold increase) assays, whereas *K. marxianus* and *L. helveticus* treatments did not result in any significant differences compared with the control group. This finding suggests that the proteolytic system of *B. subtilis* may be responsible for the production of peptides with elevated antioxidant potential (Emkani et al. 2023). Ren et al. (2025) reported that the antioxidant capacity of *B. amyloliquefaciens* SY07‐fermented mixed grains after 72 h had increased, with values of 8.64 mg TE/g DW and 3.21 mg TE/g DW for ABTS and DPPH radical scavenging capacities, respectively. Moreover, Li and Wang (2021) showed that the solid‐state fermentation of chickpeas with *B. subtilis* improved their antioxidant properties through extensive proteolysis and the release of low‐molecular‐weight peptides that exhibited higher radical‐scavenging activities than those of the control group. These findings support our observation of stronger DPPH and ABTS responses in *B. subtilis* ‐treated samples. Accordingly, the increased abundance of peptides below 15 kDa in *B. subtilis* ‐fermented CPI may contribute to the elevated antioxidant activities observed, as low‐molecular‐weight peptides have been associated with enhanced radical‐scavenging and metal‐chelating properties (Sanjukta and Rai 2016; Zhao et al. 2021).

**TABLE 2:** Antioxidant activity and total phenolic content (TPC) of control and fermented ( *B. subtilis* , *K. marxianus* and *L. helveticus* ) chickpea protein isolate (CPI) samples after 48 h of fermentation. Antioxidant capacity is represented according to 2,2‐Diphenyl‐1‐picrylhydrazyl (DPPH) assay, 2,2‐azinobis (3‐ethylbenzothiazoline‐6‐sulfonic acid) (ABTS) assay, and cupric ion reducing antioxidant capacity (CUPRAC) assay.

Treatments	DPPH (mg TE/100 mL sample)	ABTS (mM TE/mL sample)	CUPRAC (mg TE/100 mL sample)	TPC (mg GAE/100 mL sample)
CK	0.40 ± 0.02^c^	1.47 ± 0.06^b^	8.93 ± 0.58^b^	19.7 ± 1.1
F‐BS	0.75 ± 0.02^a^	2.26 ± 0.07^a^	24.76 ± 2.40^a^	21.4 ± 3.3
F‐KM	0.44 ± 0.03^c^	1.53 ± 0.02^b^	9.78 ± 0.50^b^	17.9 ± 1.1
F‐LH	0.55 ± 0.03^b^	1.41 ± 0.04^b^	8.49 ± 0.33^b^	18.4 ± 0.8

*Note:* Values are represented as means ± standard deviations (*n* = 3). ^a‐c^Different superscript letters indicate significant differences within the same column (*p* < 0.05) following one‐way ANOVA (Tukey).

Abbreviations: CK, control group (unfermented); F‐BS, CPI fermented by *B. subtilis* ; F‐KM, CPI fermented by *K. marxianus*; F‐LH, CPI fermented by *L. helveticus* .

Despite the observed differences in antioxidant capacity, the total phenolic content (TPC) remained statistically unchanged (*p* > 0.05) across all samples. Phenolic compounds are well‐recognized contributors to antioxidant activity in plant‐based substrates, and previous studies have reported positive correlations between phenolic compounds, flavonoids, or peptide content and radical‐scavenging capacity (Kwaw et al. 2018; Ren et al. 2025). However, the absence of significant changes in TPC in the present study suggests that phenolic compounds are unlikely to be the primary drivers of the enhanced antioxidant activity observed following *B. subtilis* fermentation. Instead, the increased antioxidant capacity could be more plausibly associated with the formation of low‐molecular‐weight peptides generated via microbial proteolysis. However, it should be noted that no direct characterization of peptide or phenolic profiles was performed in this study, which limits definitive conclusions regarding the underlying mechanism. Supporting this interpretation, in the study of Tonini et al. (2024), increases in ABTS radical removal, angiotensin‐converting enzyme (ACE) inhibition, and antifungal activities showed a correlation with the release of certain bioactive peptides during the fermentation of red lentil protein isolate with several LAB and some yeast strains. Peptide profiling and quantification revealed that fermentation with *Hanseniaspora uvarum* SY1 had the most promising potential to generate bioactive peptides. Similarly, after simulated in vitro gastrointestinal digestion, the solid‐state fermented chickpea flour showed increased release of small peptides and phenolic acids (e.g., protocatechuic acid, p‐hydroxybenzoic acid, γ‐aminobutyric acid, and gallic acid), which together led to higher in vitro antioxidant activity and greater bioactive potential compared to the unfermented flour (Zhou et al. 2025).

### 3.7 Antinutritional Factors (ANFs)

Antinutritional factors, particularly phytic acid and condensed tannins, are intrinsic constituents of legume seeds and are well documented to impair mineral bioavailability and protein utilization through chelation and protein–polyphenol interactions (Arbab Sakandar et al. 2023; Sharma 2021). Therefore, monitoring the levels of these compounds following microbial fermentation is essential for evaluating the nutritional upgrading of CPI. As summarized in Table 3, fermentation induced a pronounced and strain‐dependent reduction in phytic acid content, whereas condensed tannins were moderately affected. Compared to the control group (44.5 μg/100 mL), phytic acid levels decreased to 38.3 μg/100 mL in *B. subtilis* –fermented samples (14% reduction), to 30.5 μg/100 mL following *K. marxianus* fermentation (31% reduction), and markedly to 13.3 μg/100 mL after *L. helveticus* fermentation (70% reduction). These findings are consistent with those of previous studies demonstrating that microbial fermentation, particularly by LAB, is among the most effective processing strategies for phytate degradation in chickpeas and other legumes (Anaemene and Fadupin 2022; Dida Bulbula and Urga 2018). The superior efficacy of *L. helveticus* can be attributed to both direct and indirect mechanisms of phytate hydrolysis in the gut. LAB‐driven acidification lowers the pH to levels favorable for endogenous phytase activation (pH 4.0–4.6), and microbial phytases further contribute to phytate breakdown, resulting in substantial phosphorus release (Arbab Sakandar et al. 2023; S. Zhou et al. 2025). Similar magnitudes of phytic acid reduction (45%–96%) have been reported in fermented chickpea meals and other legume matrices, supporting the robustness of this mechanism across substrates and fermentation systems (Anaemene and Fadupin 2022). In contrast, yeast‐mediated fermentation (*K. marxianus*) achieved a moderate but significant decrease, which aligns with the literature indicating that yeasts primarily contribute to organic acid production rather than strong intrinsic phytase activity (Arbab Sakandar et al. 2023).

**TABLE 3:** Different antinutritional factors of the chickpea protein isolate (CPI) samples fermented for 48 h by *Bacillus subtilis* (B‐3387), *Kluyveromyces marxianus* (Y‐329), and *Lactobacillus helveticus* (B‐4526).

Treatments	Condensed tannins (mg catechin/100 mL)	Phytic acid (μg/100 mL)
CK	3.20 ± 0.25^a^	44.5 ± 0.2^a^
F‐BS	3.22 ± 0.04^a^	38.3 ± 0.6^b^
F‐KM	2.91 ± 0.13^ab^	30.5 ± 3.9^c^
F‐LH	2.68 ± 0.18^b^	13.3 ± 1.0^d^

*Note:* Values are represented as means ± standard deviations (*n* = 3). ^a‐c^Different superscript letters indicate significant differences within the same column (*p* < 0.05) following one‐way ANOVA (Tukey).

Abbreviations: CK, control group; F‐BS, CPI fermented by *B. subtilis* ; F‐KM, CPI fermented by *K. marxianus*; F‐LH, CPI fermented by *L. helveticus* .

In contrast, fermentation led to limited reductions in condensed tannins level, decreasing from 3.20 mg catechin/100 mL in the control to 2.68 mg catechin/100 mL in *L. helveticus* –fermented samples. This modest response is in agreement with earlier reports showing that tannins may be more resistant to microbial degradation than phytates, particularly when present in protein‐rich matrices where tannin–protein complexes may limit enzymatic accessibility (Dida Bulbula and Urga 2018; Sharma 2021). Nevertheless, partial tannin reduction through fermentation has been consistently observed and is considered nutritionally relevant, as even small decreases may alleviate the negative effects on protein digestibility and enzyme inhibition (Arbab Sakandar et al. 2023).

Overall, the differential responses of phytic acid and condensed tannins highlight the importance of microorganism selection in CPI fermentation. The pronounced phytate degradation achieved with *L. helveticus* underscores the potential of LAB‐based fermentation as a targeted strategy to mitigate mineral‐binding antinutritional factors, thereby enhancing the nutritional and functional quality of chickpea protein. These results corroborate the extensive literature on legume fermentation and reinforce microbial fermentation as a scalable and effective bioprocess for improving plant protein quality.

### 3.8 Correlation Analysis

**FIGURE 4:**
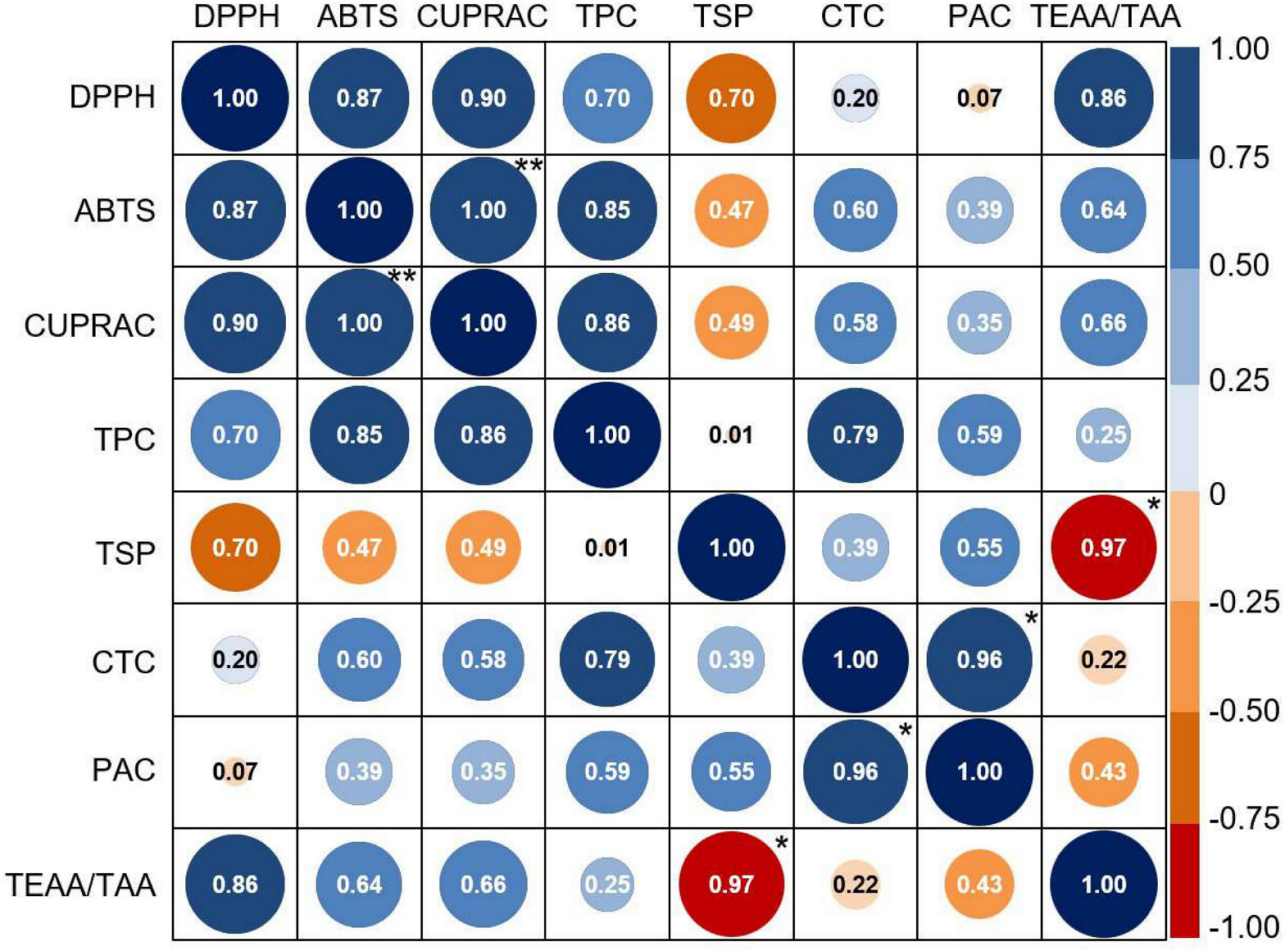
Pearson's correlation matrix showing the associations between the analyzed parameters of CPI samples. The size of the circles indicates the strength of the correlation, with larger circles indicating stronger associations and smaller circles indicating weaker relationships. The color scale bar illustrates the direction of correlation, where values approaching 1 and −1 correspond to a high positive (dark blue) and high negative (dark red) relationship between the CPI samples, respectively. Statistical significance was assessed using *p* values (*) < 0.05 and (**) < 0.01.

The biochemical properties of the fermented and control samples were analyzed using Pearson's correlation analysis to investigate the relationship between the fermentation process and the studied parameters (Figure 4). A significant positive correlation was observed between ABTS^●+^ and CUPRAC (*r* = 1.00, *p* < 0.01), and strong positive correlations were also observed between the other antioxidant assays (DPPH^●^ and CUPRAC, *r* = 0.90; DPPH^●^ and ABTS^●+^, *r* = 0.87). Given that these three assays use different principles to measure antioxidant efficacy, establishing a consistent relationship between them is challenging. TPC exhibited a moderate positive correlation with CUPRAC (*r* = 0.86, *p* > 0.05), ABTS (*r* = 0.85, *p* > 0.05), and DPPH^●^ (*r* = 0.70, *p* > 0.05) assays. Although the statistical analysis did not reveal a significant difference between the CPI samples, a moderate positive correlation was observed between phenolic compounds and antioxidant activity. The relationships among these bioactive properties were subtle yet consistent, corroborating previous observations (Saharan et al. 2020; Yakubu et al. 2022). Conversely, TSP showed a strong negative correlation with the ratio of essential amino acids (TEAA/TAA; *r *= −0.97, *p* < 0.05), and an intermediate negative correlation with the three antioxidant activities. The reduction in the content of solubilized proteins during fermentation may inversely affect the antioxidant capacity and amino acid balance, potentially reflecting proteolytic processes. Furthermore, the essential amino acid ratio demonstrated a moderate and positive correlation with DPPH (*r* = 0.86), CUPRAC (*r* = 0.66), and ABTS (*r* = 0.64) antioxidant capacities. This relationship is particularly relevant from the standpoint of nutritional physiology (Yiğit and Erekul 2023). In addition, TSP exhibited a weak association with TPC, indicating its limited contribution to phenolic compound levels. Furthermore, the condensed tannin content (CTC) was significantly positively correlated (*r* = 0.96, *p* < 0.05) with the phytic acid content (PAC). The strong correlation between these ANFs following fermentation indicates their co‐degradation by microbial enzymes, as the fermentation conditions promote simultaneous reductions in both factors (Egounlety and Aworh 2003; Jeyakumar and Lawrence 2022).

## 4 Conclusion

This study demonstrates that microbial fermentation is a powerful and versatile tool for tailoring the nutritional and functional quality of CPIs. Distinct microorganism‐dependent modifications were revealed by applying lactic acid bacteria, *Bacillus*, and the yeast under identical conditions, highlighting that microbial selection is a decisive factor in designing fermented plant protein ingredients. Among the tested strains, *Bacillus subtilis* emerged as the most effective modulator of protein quality, inducing extensive proteolysis, enriching the essential amino acid profile, and generating strong antioxidant and antimicrobial activities, most likely through the formation of low‐molecular‐weight peptides. In contrast, *Lactobacillus helveticus* primarily improved nutritional quality through the efficient reduction of anti‐nutritional factors, whereas *Kluyveromyces marxianus* exerted more moderate structural and functional effects. However, this study is limited to physicochemical and analytical assessments. Future studies should include peptide and phenolic compound profiling, as well as in vitro gastrointestinal digestion and in vivo validation, to better assess the speculated functional and health‐promoting potential of fermented CPI samples. Additionally, the applicability of these fermented CPI ingredients in food systems and their industrial feasibility should be further investigated.

In conclusion, these results highlight fermentation as a strain‐specific bioprocess that can be strategically exploited to modulate protein properties. From a practical formulation perspective, these fermented chickpea ingredients hold significant potential as multifunctional, clean‐label components in the food industry. Due to their enhanced antioxidant and potent antimicrobial activities, they can be directly incorporated into the formulations of functional cereal bars and baked snacks to naturally extend shelf‐life and replace synthetic preservatives. Furthermore, the substantial reduction in antinutritional factors, combined with improved protein digestibility, makes these ingredients highly suitable for fortifying plant‐based meat or dairy alternatives, infant formulas, and sports nutrition supplements where both high protein quality and clean digestibility are strictly required.

## Author Contributions

**Buse Bıyıklı:** investigation, visualization, writing – review and editing, data curation. **Cansu Yay:** investigation. **Bilgen Özsoy:** investigation, data curation, visualization, writing – original draft. **Müge İşleten Hoşoğlu:** project administration, writing – review and editing, conceptualization, supervision. **Emine Aytunga Arık Kibar:** data curation, writing – review and editing, funding acquisition, project administration, conceptualization. **Özlem Aslan:** investigation, data curation, visualization, writing – original draft.

## Funding

This study was financially supported by the European Union's Horizon Europe research and innovation programme (Excel4Pro, Grant number: 101186662).

## Disclosure

Declaration of Generative AI and AI‐Assisted Technologies in the Manuscript Preparation Process: This study employed ChatGPT and Perplexity exclusively for linguistic refinement to improve the clarity and readability of the manuscript. After using these tools, the authors reviewed and revised the content as necessary and take full responsibility for the published content.

## Conflicts of Interest

The authors declare no conflicts of interest.

## Data Availability

The data that support the findings of this study are available on request from the corresponding author. The data are not publicly available due to privacy or ethical restrictions.

## References

[fsn372099-bib-0001] Alrosan, M. , T.‐C. Tan , W. Y. Koh , A. M. Easa , S. Gammoh , and M. H. Alu'datt . 2023. “Overview of Fermentation Process: Structure‐Function Relationship on Protein Quality and Non‐Nutritive Compounds of Plant‐Based Proteins and Carbohydrates.” Critical Reviews in Food Science and Nutrition 63, no. 25: 7677–7691. 10.1080/10408398.2022.2049200. 35266840

[fsn372099-bib-0002] Anaemene, D. , and G. Fadupin . 2022. “Anti‐Nutrient Reduction and Nutrient Retention Capacity of Fermentation, Germination and Combined Germination‐Fermentation in Legume Processing.” Applied Food Research 2, no. 1: 100059. 10.1016/j.afres.2022.100059.

[fsn372099-bib-0003] Ankolekar, C. , M. Pinto , D. Greene , and K. Shetty . 2012. “In Vitro Bioassay Based Screening of Antihyperglycemia and Antihypertensive Activities of *Lactobacillus acidophilus* Fermented Pear Juice.” Innovative Food Science & Emerging Technologies 13: 221–230. 10.1016/j.ifset.2011.10.008.

[fsn372099-bib-0004] Apak, R. , K. Güçlü , M. Özyürek , B. Bektaşoǧlu , and M. Bener . 2010. “Cupric Ion Reducing Antioxidant Capacity Assay for Antioxidants in Human Serum and for Hydroxyl Radical Scavengers.” In Methods in Molecular Biology (Clifton, N.J.), vol. 594, 215–239. Humana Press. 10.1007/978-1-60761-411-1_15. 20072920

[fsn372099-bib-0005] Arbab Sakandar, H. , Y. Chen , C. Peng , X. Chen , M. Imran , and H. Zhang . 2023. “Impact of Fermentation on Antinutritional Factors and Protein Degradation of Legume Seeds: A Review.” Food Reviews International 39, no. 3: 1227–1249. 10.1080/87559129.2021.1931300.

[fsn372099-bib-0006] Arik Kibar, E. A. , and Ö. Aslan . 2024. “Ultrasound‐Assisted Extraction of Chickpea Proteins and Their Functional and Technological Properties.” Food Technology and Biotechnology 62, no. 4: 488–500. 10.17113/ftb.62.04.24.8502. 39830869 PMC11740741

[fsn372099-bib-0007] Brand‐Williams, W. , M. E. Cuvelier , and C. Berset . 1995. “Use of a Free Radical Method to Evaluate Antioxidant Activity.” LWT ‐ Food Science and Technology 28, no. 1: 25–30. 10.1016/S0023-6438(95)80008-5.

[fsn372099-bib-0008] Celiktas, O. Y. , E. E. H. Kocabas , E. Bedir , F. V. Sukan , T. Ozek , and K. H. C. Baser . 2007. “Antimicrobial Activities of Methanol Extracts and Essential Oils of *Rosmarinus officinalis* , Depending on Location and Seasonal Variations.” Food Chemistry 100, no. 2: 553–559. 10.1016/j.foodchem.2005.10.011.

[fsn372099-bib-0009] Chotinu‐kul, T. , G. Theeragool , and D. Chonudomkul . 2025. “Improvement of Protein Quality and Reduction of Anti‐Nutritional Factors in Soybean Meal by Solid‐State Fermentation With *Bacillus siamensis* MH03.” Biotechnology Reports 48: e00915. 10.1016/j.btre.2025.e00915. 40894330 PMC12398142

[fsn372099-bib-0046] De Melo Pereira, G. V. , D. P. De Carvalho Neto , A. C. D. O. Junqueira , et al. 2020. “A Review of Selection Criteria for Starter Culture Development in the Food Fermentation Industry.” Food Reviews International 36, no. 2: 135–167. 10.1080/87559129.2019.1630636.

[fsn372099-bib-0010] Di Francesco, A. , M. A. De Santis , A. Lanzoni , et al. 2024. “Mass Spectrometry Characterization of the SDS‐PAGE Protein Profile of Legumins and Vicilins From Chickpea Seed.” Food 13, no. 6: 887. 10.3390/foods13060887. PMC10969193 38540876

[fsn372099-bib-0011] Dida Bulbula, D. , and K. Urga . 2018. “Study on the Effect of Traditional Processing Methods on Nutritional Composition and Anti Nutritional Factors in Chickpea ( *Cicer arietinum* ).” Cogent Food & Agriculture 4, no. 1: 1422370. 10.1080/23311932.2017.1422370.

[fsn372099-bib-0012] Du, Q. , H. Li , M. Tu , et al. 2024. “Legume Protein Fermented by Lactic Acid Bacteria: Specific Enzymatic Hydrolysis, Protein Composition, Structure, and Functional Properties.” Colloids and Surfaces B: Biointerfaces 238: 113929. 10.1016/j.colsurfb.2024.113929. 38677155

[fsn372099-bib-0013] Egounlety, M. , and O. C. Aworh . 2003. “Effect of Soaking, Dehulling, Cooking and Fermentation With Rhizopus Oligosporus on the Oligosaccharides, Trypsin Inhibitor, Phytic Acid and Tannins of Soybean ( *Glycine max* Merr.), Cowpea ( *Vigna unguiculata* L. Walp) and Groundbean (Macrotyloma Geocarpa Harms).” Journal of Food Engineering 56, no. 2–3: 249–254. 10.1016/S0260-8774(02)00262-5.

[fsn372099-bib-0014] El Youssef, C. , P. Bonnarme , S. Fraud , A.‐C. Péron , S. Helinck , and S. Landaud . 2020. “Sensory Improvement of a Pea Protein‐Based Product Using Microbial co‐Cultures of Lactic Acid Bacteria and Yeasts.” Food 9, no. 3: 349. 10.3390/foods9030349. PMC7143830 32192189

[fsn372099-bib-0015] Emkani, M. , S. Moundanga , B. Oliete , and R. Saurel . 2023. “Protein Composition and Nutritional Aspects of Pea Protein Fractions Obtained by a Modified Isoelectric Precipitation Method Using Fermentation.” Frontiers in Nutrition 10: 1284413. 10.3389/fnut.2023.1284413. 38024383 PMC10652897

[fsn372099-bib-0016] Emkani, M. , B. Oliete , and R. Saurel . 2022. “Effect of Lactic Acid Fermentation on Legume Protein Properties, a Review.” Fermentation 8, no. 6: 244. 10.3390/fermentation8060244.

[fsn372099-bib-0017] Fan, M. , X. He , Y. Cao , et al. 2025. “Sustainable Microbial Fermentation of Plant Proteins: Potential, Biological Resources, Fermentation Mechanisms, Applications and Challenges in Food Industry.” Food Bioscience 68: 106727. 10.1016/j.fbio.2025.106727.

[fsn372099-bib-0018] Gao, Y. , M. Hu , W. Meng , et al. 2024. “Study on the Quality of Soybean Proteins Fermented by *Bacillus subtilis* BSNK‐5: Insights Into Nutritional, Functional, Safety, and Flavor Properties.” Food Chemistry 443: 138523. 10.1016/j.foodchem.2024.138523. 38286093

[fsn372099-bib-0019] Ghosh, A. , B. K. Das , A. Roy , B. Mandal , and G. Chandra . 2008. “Antibacterial Activity of Some Medicinal Plant Extracts.” Journal of Natural Medicines 62, no. 2: 259–262. 10.1007/s11418-007-0216-x. 18404337

[fsn372099-bib-0020] Grasso, N. , N. L. Lynch , E. K. Arendt , and J. A. O'Mahony . 2022. “Chickpea Protein Ingredients: A Review of Composition, Functionality, and Applications.” Comprehensive Reviews in Food Science and Food Safety 21, no. 1: 435–452. 10.1111/1541-4337.12878. 34919328

[fsn372099-bib-0021] Haug, W. , and H. Lantzsch . 1983. “Sensitive Method for the Rapid Determination of Phytate in Cereals and Cereal Products.” Journal of the Science of Food and Agriculture 34, no. 12: 1423–1426. 10.1002/jsfa.2740341217.

[fsn372099-bib-0022] Jeyakumar, E. , and R. Lawrence . 2022. “Microbial Fermentation for Reduction of Antinutritional Factors.” In Current Developments in Biotechnology and Bioengineering, 239–260. Elsevier. 10.1016/B978-0-12-823506-5.00012-6.

[fsn372099-bib-0023] Kaleda, A. , N. Sharma , K. Jakobson , I. Stulova , and S. Rosenvald . 2025. “Fermentation by Lactic Acid Bacteria During Pea Protein Isolation Reduces Undesirable Flavors and Changes Techno‐Functional Properties.” Food Chemistry 492: 145380. 10.1016/j.foodchem.2025.145380. 40628069

[fsn372099-bib-0024] Kanatt, S. R. K. A. , and A. Sharma . 2011. “Antioxidant and Antimicrobial Activity of Legume Hulls.” Food Research International 44, no. 10: 3182–3187. 10.1016/j.foodres.2011.08.022.

[fsn372099-bib-0025] Kurt, H. , M. Isleten Hosoglu , O. Guneser , and Y. Karagul‐Yuceer . 2023. “Influence of Different Bacteria Species in Chemical Composition and Sensory Properties of Fermented Spirulina.” Food Chemistry 400: 133994. 10.1016/j.foodchem.2022.133994. 36108443

[fsn372099-bib-0026] Kwaw, E. , Y. Ma , W. Tchabo , et al. 2018. “Effect of Lactobacillus Strains on Phenolic Profile, Color Attributes and Antioxidant Activities of Lactic‐Acid‐Fermented Mulberry Juice.” Food Chemistry 250: 148–154. 10.1016/j.foodchem.2018.01.009. 29412905

[fsn372099-bib-0027] Laemmli, U. K. 1970. “Cleavage of Structural Proteins During the Assembly of the Head of Bacteriophage T4.” Nature 227, no. 5259: 680–685. 10.1038/227680a0. 5432063

[fsn372099-bib-0028] Li, W. , and T. Wang . 2021. “Effect of Solid‐State Fermentation With *Bacillus subtilis* Lwo on the Proteolysis and the Antioxidative Properties of Chickpeas.” International Journal of Food Microbiology 338: 108988. 10.1016/j.ijfoodmicro.2020.108988. 33267968

[fsn372099-bib-0029] Liu, Y. , S. Zhu , Y. Li , F. Sun , D. Huang , and X. Chen . 2023. “Alternations in the Multilevel Structures of Chickpea Protein During Fermentation and Their Relationship With Digestibility.” Food Research International 165: 112453. 10.1016/j.foodres.2022.112453. 36869472

[fsn372099-bib-0030] Lowry, O. H. , N. J. Rosebrough , A. L. Farr , and R. J. Randall . 1951. “Protein Measurement With the Folin Phenol Reagent.” Journal of Biological Chemistry 193, no. 1: 265–275. 14907713

[fsn372099-bib-0031] Meinlschmidt, P. , E. Ueberham , J. Lehmann , U. Schweiggert‐Weisz , and P. Eisner . 2016. “Immunoreactivity, Sensory and Physicochemical Properties of Fermented Soy Protein Isolate.” Food Chemistry 205: 229–238. 10.1016/j.foodchem.2016.03.016. 27006235

[fsn372099-bib-0032] Nikbakht Nasrabadi, M. , A. Sedaghat Doost , and R. Mezzenga . 2021. “Modification Approaches of Plant‐Based Proteins to Improve Their Techno‐Functionality and Use in Food Products.” Food Hydrocolloids 118: 106789. 10.1016/j.foodhyd.2021.106789.

[fsn372099-bib-0033] Özsoy, B. , C. Yay , A. Yellice , et al. 2025. “Screening of Various Raw Materials and Their Protein Concentrates: Focus on Alkaline Extraction Yields and Physicochemical Characterizations.” European Food Research and Technology 251, no. 12: 4573–4588. 10.1007/s00217-025-04894-9.

[fsn372099-bib-0034] Plessas, S. , A. Fisher , K. Koureta , C. Psarianos , P. Nigam , and A. A. Koutinas . 2008. “Application of Kluyveromyces Marxianus, *Lactobacillus delbrueckii* Ssp. Bulgaricus and *L. helveticus* for Sourdough Bread Making.” Food Chemistry 106, no. 3: 985–990. 10.1016/j.foodchem.2007.07.012.

[fsn372099-bib-0035] Price, M. L. , A. E. Hagerman , and L. G. Butler . 1980. “Tannin Content of Cowpeas, Chickpeas, Pigeon Peas, and Mung Beans.” Journal of Agricultural and Food Chemistry 28, no. 2: 459–461. 10.1021/jf60228a047. 7391382

[fsn372099-bib-0036] Re, R. , N. Pellegrini , A. Proteggente , A. Pannala , M. Yang , and C. Rice‐Evans . 1999. “Antioxidant Activity Applying an Improved ABTS Radical Cation Decolorization Assay.” Free Radical Biology and Medicine 26, no. 9–10: 1231–1237. 10.1016/S0891-5849(98)00315-3. 10381194

[fsn372099-bib-0037] Ren, D. , C. Ren , J. Ren , S. Li , X. Yang , and F. Li . 2025. “Changes in Functional Activities and Volatile Flavor Compounds of Fermented Mung Beans, Cowpeas, and Quinoa Started With *Bacillus amyloliquefaciens* SY07.” Food Research International 201: 115636. 10.1016/j.foodres.2024.115636. 39849731

[fsn372099-bib-0038] Saharan, P. , P. K. Sadh , S. Duhan , and J. S. Duhan . 2020. “Bio‐Enrichment of Phenolic, Flavonoids Content and Antioxidant Activity of Commonly Used Pulses by Solid‐State Fermentation.” Journal of Food Measurement and Characterization 14, no. 3: 1497–1510. 10.1007/s11694-020-00399-z.

[fsn372099-bib-0039] Sahin, B. , M. I. Hosoglu , O. Guneser , and Y. Karagul‐Yuceer . 2022. “Fermented Spirulina Products With Saccharomyces and Non‐ Saccharomyces Yeasts: Special Reference to Their Microbial, Physico‐Chemical and Sensory Characterizations.” Food Bioscience 47: 101691. 10.1016/j.fbio.2022.101691.

[fsn372099-bib-0040] Salman, S. , G. Öz , R. Felek , A. Haznedar , T. Turna , and F. Özdemir . 2022. “Effects of Fermentation Time on Phenolic Composition, Antioxidant and Antimicrobial Activities of Green, Oolong, and Black Teas.” Food Bioscience 49: 101884. 10.1016/j.fbio.2022.101884.

[fsn372099-bib-0041] Sanjukta, S. , and A. K. Rai . 2016. “Production of Bioactive Peptides During Soybean Fermentation and Their Potential Health Benefits.” Trends in Food Science & Technology 50: 1–10. 10.1016/j.tifs.2016.01.010.

[fsn372099-bib-0042] Sharma, A. 2021. “A Review on Traditional Technology and Safety Challenges With Regard to Antinutrients in Legume Foods.” Journal of Food Science and Technology 58, no. 8: 2863–2883. 10.1007/s13197-020-04883-8. 34294949 PMC8249542

[fsn372099-bib-0043] Shi, Y. , A. Singh , D. D. Kitts , and A. Pratap‐Singh . 2021. “Lactic Acid Fermentation: A Novel Approach to Eliminate Unpleasant Aroma in Pea Protein Isolates.” LWT 150: 111927. 10.1016/j.lwt.2021.111927.

[fsn372099-bib-0044] Singleton, V. L. , R. Orthofer , and R. M. Lamuela‐Raventós . 1999. “Methods in Enzymology ‐ Oxidants and Antioxidants Part A.” Methods in Enzymology 299: 152–178. 10.1016/S0076-6879(99)99017-1.

[fsn372099-bib-0045] Tonini, S. , A. Z. A. Tlais , B. D. Galli , et al. 2024. “Lentils Protein Isolate as a Fermenting Substrate for the Production of Bioactive Peptides by Lactic Acid Bacteria and Neglected Yeast Species.” Microbial Biotechnology 17, no. 1: e14387. 10.1111/1751-7915.14387. 38263855 PMC10832563

[fsn372099-bib-0047] Wang, F. , M. Wang , L. Xu , et al. 2025. “Application and Possible Mechanism of Microbial Fermentation and Enzyme Catalysis in Regulation of Food Flavour.” Food 14, no. 11: 1909. 10.3390/foods14111909. PMC12154093 40509437

[fsn372099-bib-0048] Wang, F. , Y. Zhang , L. Xu , and H. Ma . 2020. “An Efficient Ultrasound‐Assisted Extraction Method of Pea Protein and Its Effect on Protein Functional Properties and Biological Activities.” LWT 127: 109348. 10.1016/j.lwt.2020.109348.

[fsn372099-bib-0049] Yakubu, C. M. , R. Sharma , and S. Sharma . 2022. “Fermentation of Locust Bean ( *Parkia biglobosa* ): Modulation in the Anti‐Nutrient Composition, Bioactive Profile, *in Vitro* Nutrient Digestibility, Functional and Morphological Characteristics.” International Journal of Food Science & Technology 57, no. 2: 753–762. 10.1111/ijfs.15288.

[fsn372099-bib-0050] Yiğit, A. , and O. Erekul . 2023. “Antioxidant Activity and Essential Amino Acid Content of Bread Wheat ( *Triticum aestivum* L.) Varieties.” Tarım Bilimleri Dergisi 29, no. 1: 130–141. 10.15832/ankutbd.999660.

[fsn372099-bib-0051] Yousefi, N. , B. Shokrollahi Yancheshmeh , and K. V. Gernaey . 2025. “The Potential of Fermentation‐Based Processing on Protein Modification: A Review.” Food 14, no. 20: 3461. 10.3390/foods14203461. PMC12562408 41153997

[fsn372099-bib-0052] Zhang, X. , X. Sun , W. Li , et al. 2021. “In Vitro and In Vivo Antioxidant Activities of Soy Protein Isolate Fermented With *Bacillus subtilis* Natto.” Journal of Food Science and Technology 58, no. 8: 3199–3204. 10.1007/s13197-020-04823-6. 34294982 PMC8249477

[fsn372099-bib-0053] Zhao, Y.‐S. , A. S. Eweys , J.‐Y. Zhang , et al. 2021. “Fermentation Affects the Antioxidant Activity of Plant‐Based Food Material Through the Release and Production of Bioactive Components.” Antioxidants 10, no. 12: 2004. 10.3390/antiox10122004. 34943107 PMC8698425

[fsn372099-bib-0054] Zhou, G. , Q. Wang , Y. Wang , et al. 2023. “Outer Membrane Porins Contribute to Antimicrobial Resistance in Gram‐Negative Bacteria.” Microorganisms 11, no. 7: 1690. 10.3390/microorganisms11071690. 37512863 PMC10385648

[fsn372099-bib-0055] Zhou, S. , Y. Wang , Q. Hu , J. Li , J. Chen , and X. Liu . 2025. “Enhancement of Nutritional Quality of Chickpea Flour by Solid‐State Fermentation for Improvement of In Vitro Antioxidant Activity and Protein Digestibility.” Food Chemistry 468: 142418. 10.1016/j.foodchem.2024.142418. 39706118

